# Blinded by magic: eye-movements reveal the misdirection of attention

**DOI:** 10.3389/fpsyg.2014.01461

**Published:** 2014-12-17

**Authors:** Anthony S. Barnhart, Stephen D. Goldinger

**Affiliations:** ^1^Cognitive Research Lab, Department of Psychological Sciences, Northern Arizona UniversityFlagstaff, AZ, USA; ^2^Department of Psychology, Arizona State UniversityTempe, AZ, USA

**Keywords:** magic, attention, inattentional blindness, perceptual load, eye-movements, eye-tracking, covert attention

## Abstract

Recent studies (e.g., Kuhn and Tatler, 2005) have suggested that magic tricks can provide a powerful and compelling domain for the study of attention and perception. In particular, many stage illusions involve attentional misdirection, guiding the observer's gaze to a salient object or event, while another critical action, such as sleight of hand, is taking place. Even if the critical action takes place in full view, people typically fail to see it due to inattentional blindness (IB). In an eye-tracking experiment, participants watched videos of a new magic trick, wherein a coin placed beneath a napkin disappears, reappearing under a different napkin. Appropriately deployed attention would allow participants to detect the “secret” event that underlies the illusion (a moving coin), as it happens in full view and is visible for approximately 550 ms. Nevertheless, we observed high rates of IB. Unlike prior research, eye-movements during the critical event showed different patterns for participants, depending upon whether they saw the moving coin. The results also showed that when participants watched several “practice” videos without any moving coin, they became far more likely to detect the coin in the critical trial. Taken together, the findings are consistent with perceptual load theory (Lavie and Tsal, 1994).

## Introduction

Historically, magicians and scientists have always engaged in a discourse, typically leading to magicians applying the newest technological innovations for use in deceiving the masses. This was the case with Robert-Houdin's (1859) early use of electromagnetism to change the weight of a small box at the magician's will^1^. In recent years, the dynamic has shifted such that scientists are becoming interested in the techniques employed by magicians (Kuhn et al., 2008a; Macknik et al., 2008; Macknik and Martinez-Conde, 2010). There is an increasing awareness that magicians are informal cognitive scientists who continually test hypotheses outside of the sterile confines of the laboratory. The knowledge accrued through this informal experimentation can guide formal scientific theories (Raz and Zigman, 2009) as well as translate into fresh methodologies for studying phenomena in the lab (Hergovich et al., 2011).

Thus far, the most fruitful collaborative effort between these disparate groups has been in the study of attention and *inattentional blindness* (IB), the tendency for people to miss salient pieces of the environment when engaged in an attention-demanding task (Kuhn and Martinez, 2012). Magic provides an ecologically valid arena for studying IB both in well-controlled laboratory conditions (Kuhn et al., 2008b) and in conditions with more natural performance and viewing (Kuhn and Tatler, 2005). Furthermore, the collaboration is a natural fit, as magicians and scientists share similar analogies when discussing attention, most commonly speaking of the “spotlight of attention” (de Ascanio, 1964/2005; Kuhn and Martinez, 2012).

Binet (1894) was among the first to discuss IB in the context of magical performance, over 100 years before Mack and Rock (1998) coined of the term, saying:

When it is particularly important that certain peculiarities of a trick be not observed, even in the broad light, matters are so arranged that the attention of the spectators is drawn to another point at the decisive moment… The attention is thus distracted… rendering invisible a spectacle which is perfectly visible to all eyes (p. 564).

Despite this early observation, magic was not brought into the laboratory to study IB for more than a century: Kuhn and Tatler (2005) examined participants' eye movements as they viewed a live magical performance (by Kuhn) wherein appropriately deployed attention would allow viewers to detect the method underlying the magical effect. The trick began with the magician placing a cigarette into his mouth and picking up a ligher to ignite it. Just before lighting the cigarette, the magician discovers that he has mistakenly placed the unfiltered end into his mouth. He reorients the cigarette and then reveals that the cigarette lighter has vanished. Following this revelation, he snaps his fingers to show that the cigarette, too, has vanished. The disappearances of both the cigarette and the lighter are accomplished by dropping the objects into the magician's lap, however the spectator's attention is carefully choreographed so that these actions elude detection. The lighter is dropped while attention is captured by the readjustment of the cigarette, and the cigarette is dropped precisely at the moment that the disappearance of the lighter is revealed.

The primary dependent variable in Kuhn and Tatler's (2005) experiment was detection of the cigarette drop, a highly salient, moving visual stimulus against the dark background of the magician's shirt. IB was assessed through self-report. Participants were asked whether they knew how the cigarette had been made to vanish. Out of 20 participants, only two reported seeing the falling cigarette. Nevertheless, examination of eye movements revealed few differences between participants who detected the drop and those who did not. While the cigarette was falling, all participants were fixated on quite similar regions of the scene (usually the magician's hand, opening to show that the lighter had vanished). Furthermore, when allowed to view the magic trick again, although all participants detected the dropping cigarette, only four shifted their gaze to the cigarette as it was falling. Overall, participants tended to fixate the same regions during both viewings of the magic trick, suggesting that detection of the critical event depended upon the deployment of covert, not overt attention.

In a follow-up study, using better-controlled video-based stimuli of the same magic trick, Kuhn et al. (2008b) again found that IB could not be predicted by the proximity of participants' fixations to the falling cigarette. However, IB could be predicted by patterns of fixations following the critical event. Participants who detected the dropping cigarette fixated the hand that held the cigarette earlier than participants who did not detect the drop.

These studies show the potential value of studying magic in the laboratory, and they provide a strong foundation for the application of magic in the study of attention. In the current work, we hope to move beyond the early studies by addressing some of their limitations within a new methodology. First, as is often the case in IB studies, the primary dependent measure implemented in prior research using magic was self-report. In their treatise on the topic, Mack and Rock (1998) reported a high rate of IB stimulus detection in an experiment *without* an IB stimulus. That is, when participants were asked whether they had seen anything in the display aside from the distractor stimulus (to which they attended in order to perform the primary task), they often reported seeing an additional stimulus when none was present. Thus, demand characteristics are a genuine concern in this type of research. The use of magic adds a secondary concern to the self-report problem, the problem of inference. If participants feel compelled to provide a possible explanation, rather than admitting that they did not see how the cigarette disappeared, it is likely that many could infer the true method. Inference would result in these participants being incorrectly categorized as having detected the drop.

Kuhn et al. (2008b) presented a compelling case that their results were not undermined by participant inference. In addition to asking participants whether they detected how the cigarette vanish was accomplished, they asked how the lighter disappeared. None of the participants who detected the cigarette drop claimed knowledge of how the lighter was made to vanish. Had they inferred information about the cigarette, it would not have been a far leap to generalize that inference to the lighter. Using a similar magic trick, Kuhn and Findlay (2010) introduced an experimental manipulation to assess the potential for inference. In their experiment, a cigarette lighter was made to vanish in a method analogous to that used in Kuhn's previous experiments. However, Kuhn and Findlay also created a “fake” condition, wherein they digitally removed the falling cigarette lighter from the video. Thus, any detection of the dropping lighter in this condition could only be the result of inference, as there was no stimulus to detect. In the fake condition, none of the participants reported seeing how the lighter was made to vanish. However, when prompted to guess at the method, 40% of participants correctly inferred that the lighter was dropped. In the “real” condition (wherein the lighter was visibly dropped), none of the IB participants inferred the correct method. These results suggest that participants can successfully dissociate perception from inference and are generally honest in their self-reports, but it would clearly be preferable to implement methods that disallow inference in future studies.

A second limitation of previous experimental work using magic to study IB is the extremely short duration of the critical stimulus event. The dropping cigarette was visible for an average of 140 ms in Kuhn and Tatler (2005) and 240 ms in Kuhn et al. (2008b). In both experiments, the authors reported the initially surprising finding that IB could not be predicted by eye-movements while the falling cigarette was visible. This outcome becomes less surprising when one considers that it takes upwards of 150 ms to program and execute an eye-movement, even when the saccade target location is entirely predictable (Rayner, 1998). Given the relative complexity of attentional deployment under these dynamic viewing conditions, the time window of the IB stimulus was unlikely to be wide enough for fixations on the moving target to occur.

Perhaps more surprising than the inability to predict IB based upon fixations on the dropping cigarette is the finding reported by Memmert (2006) that IB in the now-famous “invisible gorilla” video from Simons and Chabris (1999) could not be predicted by the number of fixations or the absolute gaze duration on the gorilla, which was visible for 5 s. However, this surface similarity between findings from Memmert and Kuhn are qualified by substantial differences in methodology. One of the values of using magic to study IB is that the participant-interpreted narrative accompanying the magic plays the role of the primary task in more traditional IB studies. In the task from Simons and Chabris, time spent fixating the gorilla would have a detrimental effect upon one's ability to successfully perform the primary task (i.e., counting basketball passes). In Memmert's replication, there was not a reliable difference in performance on the primary task as a consequence of IB, suggesting that even though the gorilla may have transiently captured some participants' attention, they were motivated to perform well on the primary task, and did not spend extra time fixating the unique character. This focus on the primary task is the likely source of the null effect of IB on fixations to the gorilla, whereas the short duration of the IB stimulus is the likely source of the non-effect in the experiments of Kuhn and colleagues (Kuhn and Tatler, 2005; Kuhn et al., 2008b).

**Figure 1:**
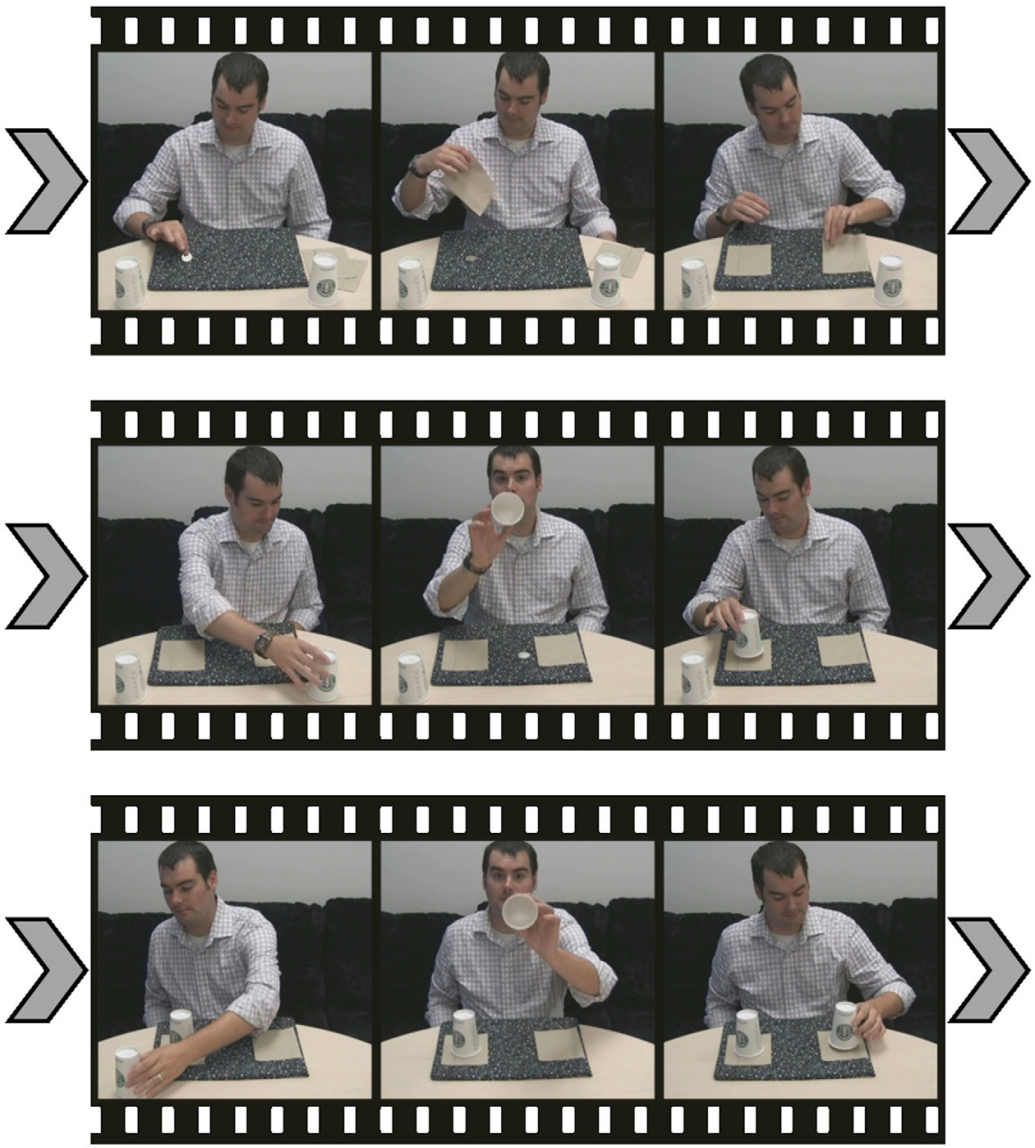
**Schematic of the actions from an experimental trial where the coin moves from under the left napkin to the right napkin**. (The contrast of the coin in the central frame has been manipulated to enhance the clarity of this graphic).

The current experiment addresses the limitations of previous IB research by using a unique methodology, borrowed from magicians, that also allows for control over a greater number of variables than previous real-world experimentation into IB. Thus, it has the potential to be a powerful tool in the study of attention and eye-movements that can be adapted to study a multitude of hypotheses. In the basic magic trick, adapted from Regal (1999), an American half-dollar coin is placed on a dark-colored placemat and is covered by a napkin. Another napkin is placed on the opposite side of the placemat. Next, an inverted cup is placed on top of each napkin, after showing the inside of each to the camera. The coin vanishes from its starting location and re-appears beneath the opposite napkin. The method of the magic trick happens in full view; see Figure 1 in *Methods* and an example video from an experimental trial in the Supplementary Materials. As the inside of the first cup is being shown to the camera, the coin visibly slides across the placemat (with a mean duration of 550 ms) to its final position beneath the second napkin. The highly salient, high-contrast coin movement often eludes detection due to misdirection provided by the action of showing the inside of the first cup to the camera.

We used a novel two-alternative forced choice method to assess IB. Participants' eye movements were monitored while they watched a video of the magic trick being performed. They were only told that they should watch the video carefully, and that they would be asked a series of questions about what they had seen afterwards. In practice, participants were never shown the revelation phase of the magic trick; they watched everything until the revelation. At the end of the video, they were queried as to the location of the coin. Thus, for participants who did not see the coin move, it felt like a very simple memory task, and they would state that the coin was at its starting position under the first napkin. However, if participants detected the coin's movement, they would say that the coin was beneath the second napkin. Participants who incorrectly identified the location of the coin were considered to be inattentionally blind.

Although we expected that our method would generally replicate findings from Kuhn and colleagues (Kuhn and Tatler, 2005; Kuhn et al., 2008b; Kuhn and Findlay, 2010), we also expected a few points of deviation. First, although (Kuhn and Tatler, 2005; Kuhn et al., 2008b) observed that eye-movements during the critical period (when the IB stimulus was visible) did not predict IB, we expected that the longer visible duration of our IB stimulus may allow eye movements to differentiate between IB and no-IB participants. Specifically, we expected no-IB participants to spend less time fixating the cup (which was shown to the camera while the coin moved across the mat) and more time fixating the space between the napkins (through which the coin moved). As with previous research, we expected that eye movements following the critical period would also indicate IB. Kuhn et al. (2008b), Kuhn and Findlay (2010) found that participants who detected the falling cigarette fixated the hand that previously held it sooner than participants who did not detect the cigarette drop. Under our methodology, we expected that participants who detected the moving coin would be more likely to fixate the space through which the coin moved or the end-point of the coin's movement sooner than participants who did not detect the coin.

The addition of a between-subjects condition in our method also allowed us to test a hypothesis derived from magicians. In their early work on IB, Mack and Rock (1998) asked participants to judge which arm of a crossbar was longer and, in critical trials, an additional stimulus was presented alongside the crossbar which served as the IB stimulus. The IB stimulus was never presented in the first trial; participants completed a few trials of the distractor task before it was presented. The structure of Mack and Rock's task resembles a structure commonly implemented in magic performance.

Sleight of hand is often designed to emulate a non-deceptive action sequence. For example, the *French Drop* sleight resembles the action of transferring a coin from one hand to the other, while actually retaining the coin in the original hand (Otero-Millan et al., 2011). To increase the odds of deception, many magicians advise that the deceptive action should be preceded by visually-similar, non-deceptive actions (i.e., the *actual* transfer of the coin from one hand to another) in order to condition the audience to accept the sleight as a normal action (de Ascanio, 1964/2005; Fitzkee, 1975; Sharpe, 1988; Lamont and Wiseman, 1999). Thus, magicians would ascribe a portion of the IB effect from Mack and Rock's work to what magic theorist Arturo de Ascanio called “conditioned naturalness” (de Ascanio, 1964/2005). By conditioning the participants to expect a certain trial structure, they become less apt to detect stimuli that do not fit within this established structure. In the Preview Condition of the present experiment, the critical trial (wherein the coin visibly moves across the mat) is preceded by three control trials wherein the coin does not move. After each trial, participants are still queried as to the location of the coin. Magicians would predict that detection of the coin's movement under these conditions would be reduced, due to the inherent conditioning of the trial structure.

However, an alternative prediction can be derived from *perceptual load* theory (Lavie, 1995; Lavie et al., 2004). This theory posits that distractor items (or the IB stimulus in Mack and Rock's, 1998, work) will be most likely to capture attention when the “perceptual load” of the primary task is low. While Lavie and Tsal (1994) admit that perceptual load is difficult to define operationally, it is rather easy to conceptualize within the current task. In the one-trial, No-Preview condition, participants were given little direction other than to watch the video with the goal of answering questions following its completion. This means that the perceptual load for the task was quite high. Participants attempted to attend to the video in its entirety, both in space and time. However, in the multiple-trial, Preview condition, the perceptual load required to successfully perform the task is reduced with each subsequent trial. Participants quickly realize that they need only encode the starting position of the coin to perform the task successfully. This reduction in perceptual load across trials 1–3 should free attentional resources to detect the coin in the critical fourth trial, reducing the IB rate.

## Methods

### Participants

Seventy-one Arizona State University undergraduates participated for partial course credit (37 in the No-Preview Condition; 34 in the Preview Condition). All participants had normal or corrected-to-normal vision.

### Materials

The magic trick was accomplished through the creation of a special mat covered in fabric with a “busy” pattern. On top of this fabric was an extra, ovular patch of the same fabric (invisible due to the pattern) connected to a string which was threaded through the mat, falling behind the table. The coin was placed on top of this extra patch of fabric. After napkins were place over the coin and over the spot on the opposite side of the mat, the inside of the first cup was shown to the camera. At the same time, the magician pulled the string beneath the table, moving the patch across the mat (taking the coin with it) to its final location beneath the opposite napkin. Figure 1 shows the sequence of events contained in one experimental trial video, wherein the coin moves from left to right.

Four videos were filmed using a Canon Vixia HV40 HD camcorder. These videos were then digitized using Windows Movie Maker and cropped to fill a screen with a 1024 × 768 aspect ratio. Two videos were created for each coin starting position (two with the coin starting on the left; two with the coin starting on the right). In each pair of videos, one was for control trials in Preview Condition wherein the coin remained in its starting position, and one was for Experimental trials in both the No-Preview and Preview Conditions wherein the coin moved across the mat. In creating the stimuli, attempts were made to maintain consistent timing of all action sequences across videos. The resulting videos all had a duration of 22 s, with the exception of one control trial in which the coin was placed on the right side of the mat, which had a duration of 21 s. Videos were presented at a rate of 30 FPS. The moving coin was visible for an average of 16.5 frames (550 ms; σ = 50) and moved in a trajectory that subtended 4° of visual angle. Stimuli were presented on a 20-inch NEC FE21111 CRT monitor (60 Hz refresh) at a viewing distance of 77 cm via SR Research Experiment Builder software running on a Dell Optiplex 755 PC (2.66 GHz, 3.25 GB RAM). Eye movements were collected monocularly at 500 Hz using an SR Research Eye-Link 1000 tracker with a spatial resolution of 0.01°.

### Procedure

This experiment was approved by the Arizona State University Human Subjects Institutional Review Board. After establishing informed consent, we calibrated participants on the tracker using a nine-point calibration procedure. The calibration procedure was repeated until the participant's average error fell below 0.5° of visual angle and no errors exceeded 1° of visual angle. Participants were told that they would view a series of short videos and answer questions after each one. The No-Preview condition contained two trials. The first trial was the experimental trial wherein the coin moved across the mat, with the starting position randomly selected for each participant. After the trial, they were queried about the coin's location and provided with accuracy feedback on their response. Accuracy on this task was used to assess IB. Regardless of their accuracy, trial two was a free-viewing trial where they watched the same video presented during trial one. In the event that they did not detect the coin's movement on trial one, they were encouraged to “figure out where they went wrong.” After trial two, they were asked whether they detected how the coin arrived at its final location. If they responded affirmatively, they were directed to describe exactly what they saw to the research assistant, who categorized them as IB or no-IB on the free-viewing trial.

The Preview condition was identical to the No-Preview condition with the exception that the experimental and free-viewing trials were preceded by three control trials wherein the coin did not move from one position to the other. The coin's position in each control video was selected randomly for each participant. Participants were queried on the coin's location after each trial, and accuracy feedback was provided.

## Results

### Inattentional blindness rates

Four participants were excluded from the No-Preview condition due to eye-tracker malfunction. Rates of IB in the experimental trial were examined in a Pearson Chi-Square analysis with factor Preview (no-preview, preview), revealing a significant effect of Preview, χ^2^_(1)_ = 9.92, *p* = 0.002. In the No-Preview condition (the 2-trial condition), 18 out of 33 participants were blind to the moving coin, while in the Preview condition (the 5-trial condition), only 6 out of 34 participants failed to detect the coin.

A second Chi-Square analysis was carried out to examine whether the direction of coin movement influenced IB. This analysis produced a null effect, χ^2^_(1)_ = 0.21, *p* = 0.65, suggesting that the videos were equivalently deceptive. When the coin moved from left to right, 39% of participants were blind to its movement, while 33% were blind to movement in the opposite direction. All further analyses collapsed across the direction of coin movement, in light of this null effect. A final Chi-Square analysis was carried out to explore rates of coin detection in the free-viewing trial. Detection rates did not differ as a function of Preview, χ^2^_(1)_ = 0.1.26, *p* = 0.26. Six participants still failed to detect the coin in the No-Preview condition, and three participants who failed to detect the coin in the experimental trial of the Preview condition also missed the coin in the free-viewing trial.

### Eye movements

**Figure 2:**
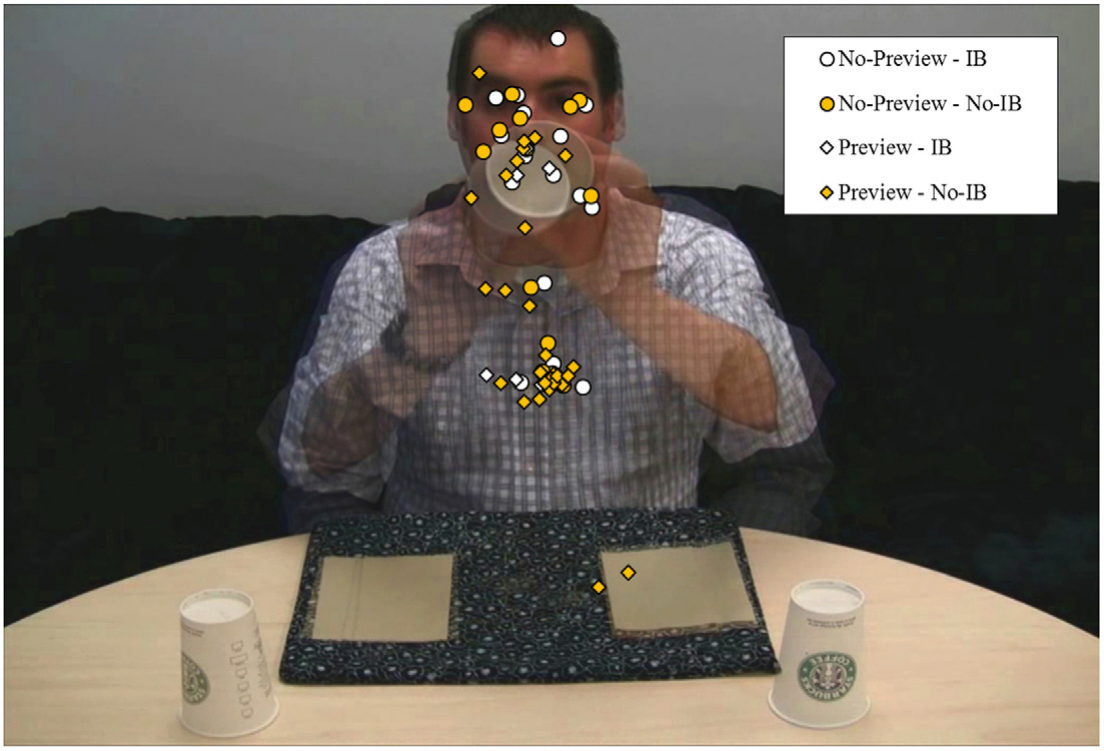
**Fixation locations at midpoint of coin's movement on the experimental trial as a function of Preview and Inattentional Blindness**. The overlay procedure used to create this graphic makes the coins invisible, as they were in subtly different positions at their temporal midpoint across the two experimental videos.

Our first analysis examined fixation distances (in pixel space) from the coin, measured at the midpoint of the coin's movement on the experimental trial. Figure 2 depicts the fixation locations of participants as a function of Preview and IB. The mean fixation distances are presented in Table 1. The Euclidean distance was calculated from the fixation coordinates sampled at the temporal midpoint of the coin's movement and the coordinates of the coin's location. These values were then analyzed in a univariate ANOVA with between-subjects factors Preview (no-preview, preview) and IB (blind, not blind). This analysis produced only a reliable effect of Preview, *F*_(1, 63)_ = 5.08, *p* = 0.03, η^2^_p_ = 0.08. The fixation positions of participants in the Preview condition were an average of 79 pixels closer to the moving coin than those in the No-Preview condition. We carried out the same analysis on fixation locations at the midpoint of the coin's movement during the free-viewing trial. On this trial, there was a marginal effect of IB, *F*_(1, 63)_ = 3.72, *p* = 0.058, η^2^_p_ = 0.06, with IB participants fixating locations farther from the moving coin than no-IB participants. There was no effect of Preview, *F*_(1, 63)_ = 1.78, *p* = 0.19, η^2^_p_ = 0.03.

**Table 1:** **Means (and Standard Deviations) for all eye-movement data**.

Variable	Preview		No-Preview	
	IB	No-IB	IB	No-IB
Fixation distance (in pixels) from moving coin on experimental trial	334 (122)	296 (123)	398 (122)	390 (125)
Fixation distance (in pixels) from moving coin on free-viewing trial	339 (224)	202 (137)	380 (156)	307 (143)
**PROBABILITY OF FIXATION DURING CRITICAL PERIOD**				
Starting napkin	0.06 (0.14)	0.03 (0.11)	0.03 (0.12)	0.00 (0.00)
End napkin	0.00 (0.00)	0.07 (0.17)	0.00 (0.00)	0.02 (0.06)
Space between napkins	0.06 (0.14)	0.22 (0.35)	0.00 (0.00)	0.13 (0.23)
Cup	0.64 (0.73)	0.36 (0.38)	0.62 (0.32)	0.35 (0.33)
Face	0.00 (0.00)	0.11 (0.26)	0.19 (0.27)	0.19 (0.29)
**PROBABILITY OF FIXATION DURING FREE-VIEWING TRIAL**				
Starting napkin	0.33 (0.29)	0.06 (0.17)	0.04 (0.10)	0.04 (0.13)
End napkin	0.17 (0.29)	0.16 (0.23)	0.00 (0.00)	0.06 (0.16)
Space between napkins	0.17 (0.29)	0.37 (0.37)	0.08 (0.13)	0.18 (0.28)
Cup	0.17 (0.29)	0.16 (0.29)	0.28 (0.31)	0.35 (0.42)
Face	0.17 (0.29)	0.05 (0.13)	0.26 (0.25)	0.07 (0.18)
**TIME TO FIXATE AFTER CRITICAL PERIOD (msec)**				
Starting napkin	1210 (1783)	2046 (1904)	1186 (1782)	1595 (1978)
End napkin	4539 (1182)	2591 (2807)	3426 (1445)	996 (1532)
Space between napkins	317 (242)	1180 (1405)	7687 (7148)	773 (765)
Face	2382 (3363)	3454 (2000)	892 (1509)	3004 (3083)

**Figure 3:**
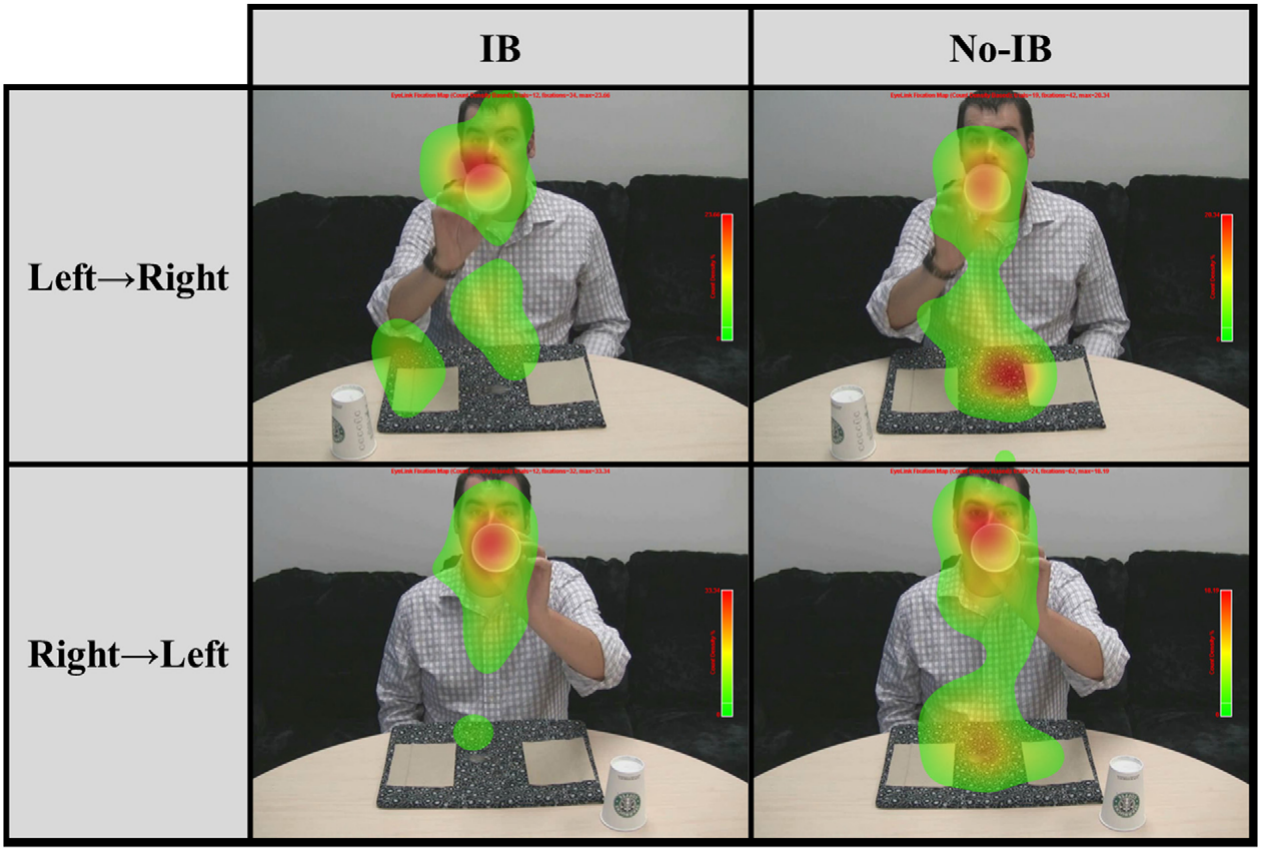
**Fixation patterns during the critical period as a function of the direction of coin movement and inattentional blindness**.

Next, we examined the proportion of fixations falling upon five different regions of interest (ROIs) during the entire 550-ms critical period when the coin was visibly moving across the screen in the IB trial: the napkin covering the coin's starting position, the napkin covering the coin's end point, the space between the napkins (through which the coin was moving), the cup which was being displayed to the camera, and the magician's face (which was partially occluded by the cup). Figure 3 depicts the pattern of fixations (shown as a heat map) during the critical period as a function of coin movement direction and IB, and Table 1 shows the probability of fixating each ROI as a function of Preview Condition and IB. We conducted a multivariate ANOVA on the proportions of fixations falling upon each ROI, with between-subjects factors Preview (no-preview, preview) and IB (blind, not blind). The omnibus MANOVA did not produce any effects related to Preview, but there was a reliable main effect of IB, *F*_(5, 59)_ = 2.41, *p* = 0.047, η^2^_p_ = 0.17. This main effect was driven by differences in two ROIs. IB participants were significantly more likely to fixate the cup during the critical period, *F*_(1, 63)_ = 7.17, *p* = 0.009, η^2^_p_ = 0.06. Furthermore, IB participants were significantly less likely to fixate the space through which the coin moved, *F*_(1, 63)_ = 4.15, *p* = 0.046, η^2^_p_ = 0.06. No other fixation patterns differed significantly as a consequence of IB.

The same analysis was applied to fixations during the critical period of the free-viewing trial, however, the outcome differed (see Table 1 for descriptive statistics). The omnibus MANOVA produced reliable main effects of Preview, *F*_(5, 59)_ = 2.38, *p* = 0.049, η^2^_p_ = 0.17, and IB, *F*_(5, 59)_ = 2.96, *p* = 0.02, η^2^_p_ = 0.20. The Preview effect was driven by differences in the probability of fixating the starting-point napkin during the critical period, *F*_(1, 63)_ = 7.26, *p* = 0.009, η^2^_p_ = 0.10. Participants in the Preview condition were more likely to fixate the starting-point napkin (*M* = 0.19) than participants in the No-Preview condition (*M* = 0.04). There was also a marginal Preview effect upon the probability of fixating the end-point napkin, *F*_(1, 63)_ = 3.45, *p* = 0.068, η^2^_p_ = 0.05. Participants in the Preview condition were more likely to fixate the end-point napkin (*M* = 0.16) than those in the No-Preview condition (*M* = 0.03). The IB effect was driven primarily by differences in the probability of fixation in two ROIs. IB participants were more likely to fixate the face, *F*_(1, 63)_ = 5.94, *p* = 0.02, η^2^_p_ = 0.09, and the coin's starting position, *F*_(1, 63)_ = 5.33, *p* = 0.02, η^2^_p_ = 0.08.

We also examined fixation patterns following the critical period. Our first analyses examined how soon, following the critical period, participants fixated each of four ROIs during the experimental trial: the napkin covering the coin's starting position, the napkin covering the coin's end position, the space between the napkins (through which the coin moved), and the performer's face. These times to fixate were tested in individual ANOVAs with between-subjects factors Preview (no-preview, preview) and IB (blind, not blind). Table 1 contains the average times to fixate each ROI. There were no reliable differences in time to fixate the starting-point napkin. However, there was a significant IB effect on time to fixate the end-point napkin, *F*_(1, 39)_ = 7.44, *p* = 0.01, η^2^_p_ = 0.16. Participants who detected the coin's movement fixated the end-point napkin 2.19 s sooner than participants who did not detect the coin's movement. Analysis of the time to fixate the space between the napkins produced two reliable main effects and a significant interaction. Participants in the Preview condition fixated the space between the napkins significantly sooner than those in the No-Preview condition, *F*_(1, 31)_ = 7.11, *p* = 0.01, η^2^_p_ = 0.19. Furthermore, participants who detected the coin's movement fixated the space between the napkins sooner than those who were inattentionally blind, *F*_(1, 31)_ = 5.37, *p* = 0.03, η^2^_p_ = 0.15. These main effects were qualified by a Preview X IB interaction, *F*_(1, 39)_ = 8.87, *p* = 0.006, η^2^_p_ = 0.22. In the No-Preview condition, participants who detected the coin's movement fixated the space between the napkins almost 7 s sooner than IB participants, but the effect flipped in the Preview condition, with IB participants fixating this space 863 ms sooner than no-IB participants. Finally, there was a significant IB effect on time to fixate the magician's face, *F*_(1, 63)_ = 5.85, *p* = 0.02, η^2^_p_ = 0.09. IB participants fixated the magician's face 1.59 s sooner than no-IB participants.

**Figure 4:**
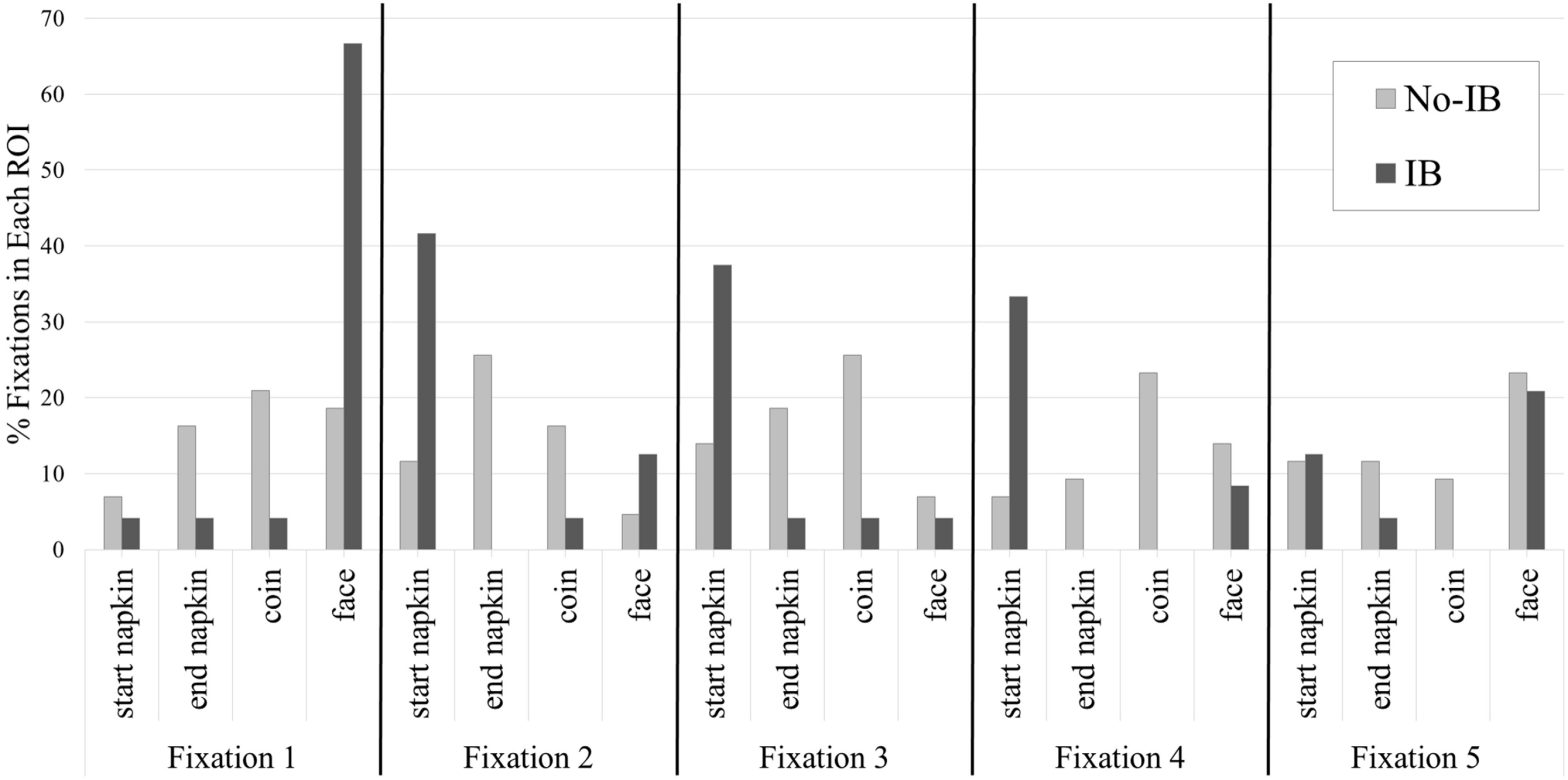
**The proportion of each of the first five fixations falling in each ROI as a function of inattentional blindness**.

**Figure 5:**
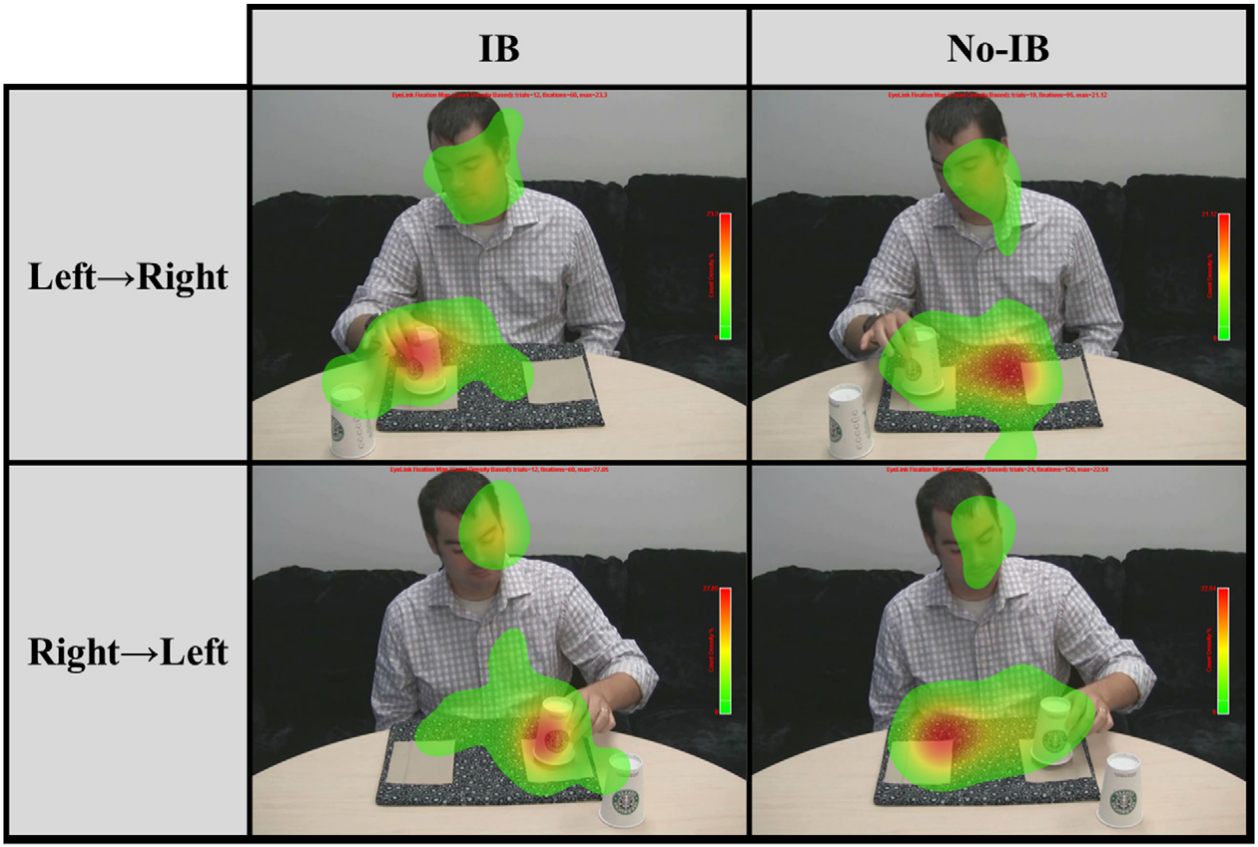
**Heatmap depicting the first five fixations following the critical period as a function of the direction of coin movement and inattentional blindness**.

We next turned to analyses of the sequence of fixations following the critical period. We performed a series of Pearson chi-square tests of independence on the first five fixations that participants made following the critical period to determine whether fixation patterns differed as a consequence of IB. The proportion of fixations falling within each ROI are shown in Figure 4, and heatmaps of the first five fixations following the critical period are in Figure 5.

The first four fixations following the critical period (but not the fifth) differed significantly, based on IB. The distribution of first fixations, χ^2^_(3)_ = 13.59, *p* = 0.004, showed that participants who were blind to the moving coin almost wholly fixated on the magician's face, while participants who detected the coin generally distributed their fixations between the endpoint of the coin's movement, the space between the napkins, and the magician's face. The distribution of second fixation landing points, χ^2^_(3)_ = 15.50, *p* = 0.001, were shifted relative to the first fixation. IB participants primarily fixated the napkin under which the coin was initially placed, whereas participants who detected the coin were primarily focused on the napkin covering the endpoint of the coin's movement and the space through which the coin moved. In the third set of fixations, χ^2^_(3)_ = 10.69, *p* = 0.01, IB participants maintained their bias to fixate the starting position napkin, while no-IB participants distributed their fixations across all ROIs, with a slight bias to fixate the space through which the coin moved. The fourth fixations, χ^2^_(3)_ = 15.57, *p* = 0.001, showed the same pattern. However, a chi-square test on the fifth set of fixations produced no effect, χ^2^_(3)_ = 2.54, *p* = 0.47: Fixation patterns at this point were no longer influenced by IB.

## Discussion

Our results replicate and extend the work of Kuhn and colleagues (Kuhn and Tatler, 2005; Kuhn et al., 2008b; Kuhn and Findlay, 2010) using a technique that improves upon prior magical methods that have been implemented in the laboratory. In the pure form of the task (the No-Preview condition), just over half the participants failed to detect a highly-salient, shiny object moving across the computer screen. This proportion was substantially reduced in the Preview condition, with the addition of three control trials without an IB stimulus. Kuhn and Tatler (2005, Kuhn et al., 2008b) observed that IB could not be predicted by fixation proximity to the IB stimulus during the critical period. As in this previous work, participants' fixation loci at the midpoint of the critical period did not predict IB. However, participants in the Preview condition tended to fixate closer to the IB event than participants in No-Preview condition. Thus, the repeated-trial structure influenced patterns of attentional deployment. While the IB rate was reduced in the Preview condition, susceptibility to IB was not influenced by participants' fixations toward the midpoint of the coin's movement. This outcome suggests differential deployment of covert attention in the Preview condition.

From their analogous result, Kuhn and Tatler (2005, Kuhn et al., 2008b) concluded that oculomotor behavior during the critical period does not predict IB. However, as already noted, their IB stimulus had a very short on-screen duration. If we expand the sampling window to include the entire 550-ms duration of the critical event, IB was signaled by participants' eye movements, unlike the outcomes reported by Kuhn and colleagues. For participants who detected the moving coin, a smaller proportion of fixations fell upon the cup (which acted as a tool for the misdirection of attention), relative to participants who did not detect the coin, and more fixations fell upon the space between the napkins. This suggests that Kuhn et al. (2008b) could not differentiate participants based on fixation patterns because of the short duration of their IB stimulus. With a longer IB stimulus (in the absence of a perceptually demanding distractor task like that of Simons and Chabris, 1999), eye movements do predict IB.

We also replicated the finding that fixation patterns after the critical period differ as a consequence of IB. Participants who detected the moving coin fixated both the space through which the coin moved and its endpoint sooner than participants who failed to detect the coin. This difference was magnified in the Preview condition, wherein no-IB participants fixated the space between the napkins almost immediately after the critical period.

Kuhn and Findlay (2010) observed that half of the participants who detected the IB stimulus in their task made up to three saccades before fixating the location where the IB stimulus appeared. Similarly, Kuhn et al. (2008b) showed that the majority of participants who detected the dropping cigarette fixated the magician's face before moving their eyes to the space previously occupied by the cigarette. This raises the question, how far beyond the critical period do fixation patterns differ as a consequence of IB? In our task, IB groups differed in the first four fixations following the critical period, but not the fifth, with IB participants showing a tendency to fixate the coin's starting position and no-IB participants showing a bias toward fixating the space through which the coin moved, or the endpoint of the its movement. Given the differences between our task and that of Kuhn and colleagues, the IB participants may have been offloading the task of remembering the coin's location by maintaining fixation on the location where they saw the coin being placed.

Beyond replicating and extending previous results, the current experiment contributes to the burgeoning “science of magic” (Kuhn et al., 2008a; Macknik et al., 2008; Macknik and Martinez-Conde, 2010) by examining a long-held intuition of magicians, the value of “conditioned naturalness” (de Ascanio, 1964/2005). In order to mask a deceptive action, magicians advise that the action it is meant to simulate should be carried out (ideally several times) prior to the deceptive action. This prior experience with the action is meant to condition the observer to accept the deceptive action sequence as natural. Under this logic, participants should have been most susceptible to IB in the Preview condition, after having been conditioned to trials devoid of deception (or, at least without an IB stimulus). However, despite identical stimuli across conditions, participants in the Preview condition were substantially *less* susceptible to IB than participants in the No-Preview condition, the single-trial condition. This outcome is predicted by an extrapolation of perceptual load theory (Lavie and Tsal, 1994; Lavie, 1995; Lavie et al., 2004). Repeated experience with the trial structure reduces the perceptual load of the task, freeing attentional resources to detect the IB stimulus in the experimental trial. While it does not refute magic's “natural conditioning” hypothesis in all situations, the present experiment deepens our understanding of the conditions under which the hypothesis may or may not be applicable, just as recent research testing illusory motion has highlighted conditions wherein joint attention fails to enhance the perception of magic (Cui et al., 2011).

Alternatively, the reduced IB that occurred with repeated trials could reflect decreased novelty of the video, or interest in the cup, over time. Participants who failed to detect the moving coin were continually engaged with the cup during the critical period, while participants who detected the coin tended to fixate the space between the napkins. Importantly this viewing pattern did not differ significantly as a function of Preview condition. Thus, it seems that the *scope* of attention differed by Preview condition, rather than its *placement*, a conclusion that also aligns with perceptual load theory. Further research could easily disentangle these alternative interpretations through manipulation of interest in the cup, itself. In the current stimulus, the magicians gazes into the cup before presenting it to the camera, thus increasing interest in the cup. Removing this gaze component may reduce IB rates, if the novelty hypothesis is correct.

The ability to carry out this simple manipulation highlights an attractive feature of the current method, which offers a versatile tool for the study of IB under conditions of (almost) natural viewing. Although a coin was used as the IB stimulus in the current experiment, the method is quite flexible (e.g., the IB stimulus could be any object small enough to fit upon the sliding patch of fabric). In addition, the magician retains full control over many variables that are relevant to IB, including the speed and direction of the IB stimulus movement and social cues employed to misdirect attention. As such, the current method allows for re-examination of many variables from Mack and Rock (1998), using a framework that better emulates visual perception and attention in the real world.

The present task can also be adapted to address recent critiques of the IB/attentional misdirection literature. Memmert (2010) argued for an empirical dissociation between IB (i.e., Simons and Chabris', 1999, “Invisible Gorilla” experiment) and attentional misdirection (i.e., Kuhn and Tatler's, 2005, vanishing cigarette) paradigms, citing four major distinctions between the typical experimental protocols. One of his criticisms was that IB tasks typically implement a full-attention control trial, whereas attentional misdirection tasks do so inconsistently or ineffectively. Memmert argued that control trials in the IB literature ensure the visibility of the IB stimulus in the absence of the attention-demanding primary task, and that it is impossible to create an analogous situation in an attentional misdirection task because the attention-demanding “primary task” is the inherent narrative of the magical presentation that participants use to guide their attention. In the current experiment, we implemented just such a control trial (the free-viewing trial). Although not perfectly analogous to the control trial in IB experiments, our free-viewing trial allowed participants to refocus their attention toward relevant stimuli and away from misdirecting stimuli. Consequently, IB was greatly reduced in these trials, and eye-movement patterns changed substantially from the experimental trial.

The current task's flexibility also allows for manipulations to address Memmert's (2010) three other critiques. A distractor task (stimuli appearing within the cups) can easily be added to the video to increase participants' attentional workload. The magical methodology employed to move the item from one location to another can be adapted such that the moving object is not the object that was originally covered with a napkin (e.g., a copper coin moves across the mat after a silver coin was placed beneath a napkin). Thus, the identity of the IB stimulus would not be foreshadowed or integral to the narrative of the presentation, unlike the stimulus in most attentional misdirection tasks.

Finally, the task reported here can be adapted to explore larger questions associated with the relationship between eye-movements and attention. Paradoxically, many prior experiments have failed to find differences in eye-movements during the critical period that would predict IB (Kuhn and Tatler, 2005; Memmert, 2006; Kuhn et al., 2008b; Kuhn and Findlay, 2010). These researchers have invoked *covert attentional deployment* to explain these findings. As the name implies, covert attention is difficult to measure. However, some researchers have suggested that microsaccades, small fixational eye-movements, may point to the locus of covert attention (Hafed and Clark, 2002; Engbert and Kliegl, 2003; Hafed et al., 2011). By adding a distractor task as outlined earlier, the current paradigm could become a multi-trial divided attention task wherein IB (as measured by detection of the moving coin) can be assessed as a function of microsaccade amplitude and direction.

### Conflict of interest statement

The authors declare that the research was conducted in the absence of any commercial or financial relationships that could be construed as a potential conflict of interest.

## Footnotes

^1^Interestingly, Robert-Houdin's demonstration was also credited as the only use of magic as a means to preemptively diffuse a war, when he used his magic to “weaken” one of the soldiers from the opposing army.
